# Scavenging Capacity of Marine Carotenoids against Reactive Oxygen and Nitrogen Species in a Membrane-Mimicking System

**DOI:** 10.3390/md10081784

**Published:** 2012-08-20

**Authors:** Eliseu Rodrigues, Lilian R. B. Mariutti, Adriana Z. Mercadante

**Affiliations:** Department of Food Science, Faculty of Food Engineering, University of Campinas (UNICAMP), Rua Monteiro Lobato, 80, CEP 13083-862, Campinas, São Paulo 13083-970, Brazil; Email: eliquimica2007@gmail.com (E.R.); lilianmariutti@gmail.com (L.R.B.M.)

**Keywords:** liposomes, astaxanthin, carotenoids, ROS, RNS

## Abstract

Carotenoid intake has been associated with the decrease of the incidence of some chronic diseases by minimizing the *in vivo* oxidative damages induced by reactive oxygen (ROS) and nitrogen species (RNS). The carotenoids are well-known singlet oxygen quenchers; however, their capacity to scavenge other reactive species, such as peroxyl radical (ROO^•^), hydroxyl radical (HO^•^), hypochlorous acid (HOCl) and anion peroxynitrite (ONOO^−^), still needs to be more extensively studied, especially using membrane-mimicking systems, such as liposomes. Moreover, the identification of carotenoids possessing high antioxidant capacity can lead to new alternatives of drugs or nutritional supplements for prophylaxis or therapy of pathological conditions related to oxidative damages, such as cardiovascular diseases. The capacity to scavenge ROO^•^, HO^•^, HOCl and ONOO^−^ of seven carotenoids found in marine organisms was determined in liposomes based on the fluorescence loss of a fluorescent lipid (C_11_-BODIPY^581/591^) due to its oxidation by these reactive species. The carotenoid-bearing hydroxyl groups were generally more potent ROS scavengers than the carotenes, whilst β-carotene was the most efficient ONOO^−^ scavenger. The role of astaxanthin as an antioxidant should be highlighted, since it was a more potent scavenger of ROO^•^, HOCl and ONOO^−^ than α-tocopherol.

## 1. Introduction

Carotenoids are yellow to red fat-soluble pigments found in plants, microorganisms and animals [1,2,3,4]. Animals do not synthesize carotenoids *de novo*, and thus, those found in animals are either directly accumulated from food or partly modified through metabolic reactions. Among the 750 carotenoids found in nature identified so far, more than 250 are of marine origin and show a great structural diversity. In particular, except for neoxanthin and its derivatives, allenic carotenoids and all acetylenic carotenoids, such as fucoxanthin, are originated from marine animals and seaweeds [4]. 

Evidence from epidemiological studies and some supplementation human trials have associated the carotenoid intake with the decrease of the incidence of some chronic diseases [5,6], such as cardiovascular diseases and some types of cancer. This effect is hypothetically attributed to the antioxidant properties of the carotenoids, which minimize the *in vivo* oxidative damages induced by reactive oxygen species (ROS) and reactive nitrogen species (RNS) [7,8].

ROS and RNS are products of the normal cellular metabolism and they are well recognized for playing a dual role in living systems once their effects can be either harmful or beneficial [9]. At moderate concentrations, ROS and RNS can be involved in cellular responses to pathogens; however, some events, such as infections, can induce an overproduction of ROS and RNS that can either play a role in combating the invading organism or cause damage in the organism cell components and tissue injuries: a situation named oxidative stress [10]. 

The antioxidant properties of the carotenoids have been largely studied *in vitro* due to the complexity of *in vivo* systems. The methods to determine the antioxidant capacity of carotenoids generally use a homogenous system and measure the capacity of the carotenoids to quench singlet oxygen (^1^O_2_) [11] and to scavenge either peroxyl radicals (ROO^•^) [12,13,14] or non-biological radicals, such as ABTS [14,15]. Despite the fact that the homogeneous systems used to assay the antioxidant capacity do not resemble the cellular environment, the results obtained using these systems are often misguidedly extrapolated to possible effects on living organisms. Living organisms are extremely complex functional systems that are made up of, at a minimum, many thousands of biomolecules in an environment that is spatially organized by membranes. In this sense, the use of liposomes as mimic systems of membranes to evaluate the antioxidant capacity of bioactive compounds has been stimulated due to the similarities between the bilayer structure of the liposomes and the lipid fraction of cell membranes [16]. In fact, the literature reports the capacity of some carotenoids to scavenge ROO^•^ in liposomes [12,17,18]; however, data on the capacity of carotenoids in liposomes to scavenge other reactive species, such as hydroxyl radicals (HO^•^), hypochlorous acid (HOCl) and peroxynitrite anions (ONOO^−^), are scarce or inexistent. 

The identification of potent marine carotenoids as ROS and RNS scavengers can lead to new alternatives of drugs or nutritional supplements for prophylaxis or therapy for pathological conditions related to oxidative damages, such as cardiovascular diseases and some types of cancer, improving human healthcare. In the present study, the antioxidant capacities of seven carotenoids commonly present in marine sources against ROS and RNS of biological relevance, namely ROO^•^, HO^•^, HOCl and ONOO^−^, were determined in membrane-mimic systems (liposomes). Furthermore, this is the first time that the capacity of carotenoids to scavenge HOCl and ONOO^−^ in liposomes is reported.

## 2. Results

**Figure 1:**
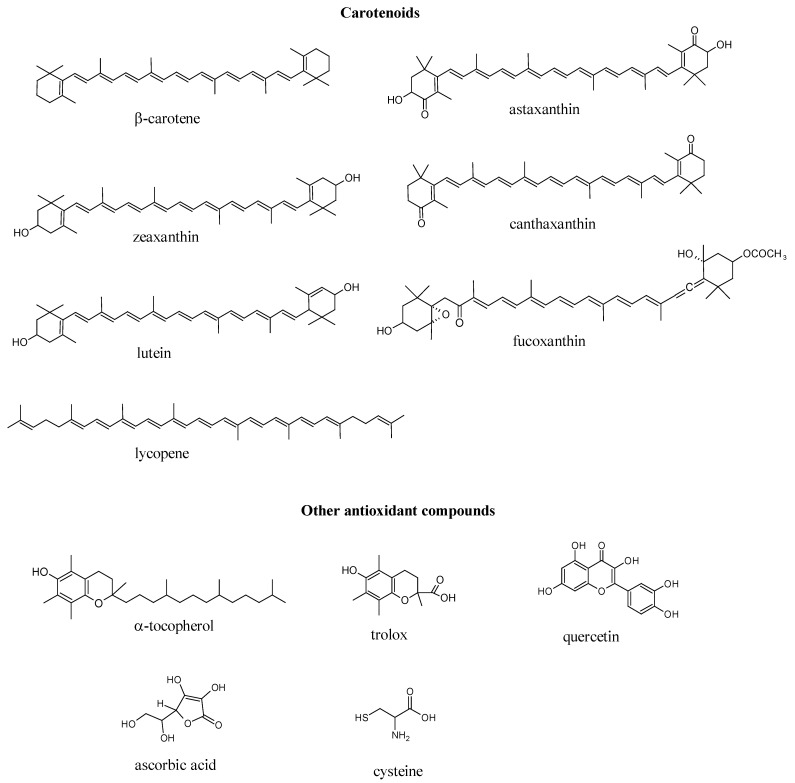
Structure of the marine carotenoids and other antioxidant compounds.

The capacity to scavenge ROO^•^, HO^•^, HOCl and ONOO^−^ of seven marine carotenoids in liposomes, *i.e.*, fucoxanthin, β-carotene, lycopene, astaxanthin, canthaxanthin, zeaxanthin and lutein (Figure 1), is shown in Table 1. To establish a comparison with other compounds widely known to possess antioxidant capacity, α-tocopherol, trolox, quercetin, ascorbic acid and cysteine were also analyzed (Table 1). Among the tested carotenoids, astaxanthin was the most efficient ROS scavenger, whilst β-carotene was the most potent RNS scavenger. Scavenging capacities were calculated considering as reference: trolox (1.00) for ROO^•^ and HO^•^, cysteine (1.00) for HOCl and ascorbic acid (1.00) for ONOO^−^.

**Table 1:** Peroxyl radical (ROO^•^), hydroxyl radical (HO^•^), hypochlorous acid (HOCl) and peroxynitrite anion (ONOO^−^) scavenging capacity of carotenoids and other compounds incorporated into liposomes, as well as partition ratio values.

Compound	Scavenging Capacity ^a^				log *P* ^b^
	ROO^•^	HO^•^	HOCl	ONOO^−^	
β-carotene	0.14	0.71	NA	1.02	14.76 ± 0.43
zeaxanthin	0.56	1.41	3.87	0.77	10.92 ± 0.45
lutein	0.60	0.97	4.81	0.78	11.52 ± 0.46
lycopene	0.08	0.35	0.40	0.31	14.53 ± 0.45
fucoxanthin	0.43	1.18	6.26	NA	7.30 ± 0.65
canthaxanthin	0.04	0.28	0.10	NA	9.53 ± 0.45
astaxanthin	0.64	1.66	9.40	0.73	8.24 ± 0.59
α-tocopherol	0.48	1.77	NA	0.37	10.96 ± 0.35
trolox	**1.00**	**1.00**	NA	NA	2.46 ± 0.36
quercetin	0.84	1.42	5.63	0.97	1.99 ± 1.08
ascorbic acid	NA ^c^	NA	0.41	**1.00**	−2.78 ± 0.42
cysteine	0.04	NA	**1.00**	0.02	0.08 ± 0.32

The values are the mean of two independent experiments. ^a^ The values of the ROO^•^ and HO^•^ scavenging capacity were calculated as equivalent to trolox, HOCl was calculated as equivalent to cysteine and ONOO^−^ was calculated as equivalent to ascorbic acid; ^b^ Partition ratio value of the antioxidant compound between water and octanol, calculated using the ACD/ChemSketch Freeware; ^c^ NA: no activity was found within the tested concentrations.

The xanthophylls, astaxanthin, lutein and zeaxanthin presented similar capacities to scavenge ROO^•^, which were higher than those of the known antioxidants α-tocopherol, ascorbic acid and cysteine, but were less effective than quercetin and trolox (Table 1). On the other hand, canthaxanthin presented the lowest ROO^•^ scavenging capacity among all the evaluated carotenoids.

The best scavenger of HO^•^ was astaxanthin, followed by zeaxanthin and fucoxanthin (Table 1). Astaxanthin was 66% and 17% more potent than trolox and quercetin, respectively, but only 6% less potent than α-tocopherol. Once more, canthaxanthin presented the lowest HO^•^ scavenging capacity among the evaluated carotenoids. 

With the exception of β-carotene, all the other carotenoids were able to scavenge HOCl. Moreover, α-tocopherol and trolox were also not able to scavenge HOCl. Astaxanthin, followed by fucoxanthin, lutein and zeaxanthin, presented the highest HOCl scavenging capacities among the carotenoids. Canthaxanthin presented the lowest HOCl scavenging capacity among the carotenoids, being 94-fold less efficient than astaxanthin. Lycopene also presented low capacity to scavenge HOCl, which was similar to that of ascorbic acid.

Apart from canthaxanthin, fucoxanthin, trolox and cysteine, all the other compounds were able to scavenge ONOO^−^. The β-carotene was the best ONOO^−^ scavenger, showing scavenging capacity similar to those of quercetin and ascorbic acid.

## 3. Discussion

Carotenoids, which are well-known singlet oxygen quenchers [11], can also scavenge other ROS and RNS. Currently, it is known that the antioxidant properties of the carotenoids in homogenous systems are closely related to their chemical structure, including aspects such as the number of conjugated double bonds, type of structural end-groups, and oxygen-containing substituents [13,17,19]. In these systems, the structure activity relationship shows that the structural characteristics of the carotenoids influence both the reactivity towards the ROS and RNS and the stability of the radicals formed after the reaction with ROS and RNS [19]. In liposomes, besides these factors, the carotenoid structure also determines the distribution and orientation of the carotenoid molecules inside the membrane, which directly affects the antioxidant capacity of the carotenoids [20]. The exact location of the carotenoids in the liposomes is still the object of study. In general, the polyene chain of the carotenoids is located in the hydrophobic core of the membrane and the carotenes display a certain orientational freedom with respect to the membrane, whilst the xanthophyll polar end-groups tend to form hydrogen bonds with the lipid membrane head-groups and water, since they are located at the membrane interface close to the axis normal to the plane of the bilayer [20]. The effect of the location and orientation of the carotenoid inside the membrane on the antioxidant capacity can be clearly observed when comparing the order of the antioxidant capacity of two carotenoids in homogeneous systems and in liposomes. For instance, among 15 carotenoids analyzed in homogeneous systems, lycopene (carotene) and lutein (xanthophyll, having two hydroxyl groups) presented the highest and the lowest scavenge capacities of ROO^•^, respectively [13]. However, in the present study, when these carotenoids were incorporated into liposomes, an opposite behavior occurred and lutein showed to be a much better ROO^•^ scavenger than lycopene. The influence of the carotenoid localization was also observed in spray-dried microcapsules of carotenoids [21,22] and in oil-in-water emulsions containing carotenoids [23]. Moreover, the hydrophilic or hydrophobic nature of the reactive species analyzed also plays an important role in the antioxidant capacity of carotenoids [17].

In the present study, the carotenoid-bearing hydroxyl groups were generally more potent ROS (ROO^•^, HO^•^ and HOCl) scavengers than the more hydrophobic carotenes. This fact indicates that carotenes are most probably in a position to intercept hydrophilic ROS entering the membrane from the aqueous phase since they are located in the hydrophobic inner core of the bilayer, whilst the carotenoids with hydroxyl end-groups span the bilayer with their end-groups located near to the hydrophobic-hydrophilic interface where the ROS attack first occurs. On the other hand, a carotene, specifically β-carotene, was the most efficient scavenger of ONOO^−^, most probably because the reactive form of ONOO^−^ in neutral pH is peroxynitrous acid (ONOOH), which is able to diffuse until the hydrophobic inner core of the bilayer, where it can interact with the carotenoids present in this site [24]. 

The structures of trolox and α-tocopherol are very similar (Figure 1); the alkyl side chain of α-tocopherol is replaced by a carboxyl group in trolox, increasing the polarity, but not modifying the phenolic hydroxyl group involved in the antioxidant mechanism of both α-tocopherol and trolox. In fact, in homogeneous systems, trolox and α-tocopherol usually present similar antioxidant capacities, e.g., against the ABTS [25] and DPPH radicals [26] and ROO^•^ [13], suggesting that the scavenging capacity of trolox and α-tocopherol comprises the donation of the phenolic hydrogen or electron transfer [27]. However, trolox and α-tocopherol presented distinct behaviors against the studied reactive species in the liposomes. α-Tocopherol was more potent than trolox as a ONOO^−^ and HO^•^ scavenger, whilst trolox showed a better antioxidant capacity than α-tocopherol against ROO^•^. These evidences suggest that the polarity of these molecules directly affects their antioxidant capacity, probably due to its influence on the distribution and orientation positioning of the antioxidant molecules inside the membrane, which affects the antioxidant capacity of the carotenoids. Recently, our research group observed similar behavior in gum Arabic and maltodextrin microcapsules containing trolox and α-tocopherol [21,22]. 

The interaction of a compound with biomembranes is strongly related to its lipophilicity, which can be expressed as the partition ratio (log *P*), which is the ratio between the compound concentration in each of the two phases of an immiscible mixture. Considering water as hydrophilic and octanol as lipophilic solvents, in the present study, the log *P* values were calculated (Table 1) and used to inflict the position of the carotenoid in the lipid membrane and its influence on the capacity to scavenge the ROS and RNS. Interestingly, a negative correlation was found between the log *P* values and the capacity of the studied carotenoids to scavenge ROS, ROO^•^ (*R* = −0.50, *p* = 0.25), HO^•^ (*R* = −0.54, *p* = 0.21) and HOCl (*R* = −0.64, *p* = 0.18); however, a positive correlation was found for RNS scavenging capacity, ONOO^−^ (*R* = 0.47, *p* = 0.29). This fact means that the more affinity a carotenoid has for the hydrophilic phase (lower log *P* values), the more potent a scavenger of ROS it is, whilst the contrary occurs for RNS. 

The reactive species HO^•^ and ROO^•^ are of particular interest because of their prominent role in lipid peroxidation, which has been related to the development of atherosclerosis and other cardiovascular diseases [10]. The HO^•^ shows a very short *in vivo* half-life of approximately 10^−9^ s, thus it is a very dangerous radical due to its high reactivity [28], and when produced *in vivo*, HO^•^ reacts closely to its site of formation, where it can initiate the lipid peroxidation by abstracting an allylic hydrogen from an unsaturated fatty acid generating ROO^•^. Chain-breaking antioxidants can interrupt these reactions by scavenging the lipid ROO^•^. The best known chain-breaking antioxidants are α-tocopherol, ascorbic acid and phenolic compounds, which scavenge HO^•^ and ROO^•^ by donating a hydrogen atom, forming a lipid hydroperoxide and a resonance-stabilized antioxidant radical [27]. Three mechanisms are proposed for scavenging of ROO^•^ and HO^•^ by carotenoids, *i.e.*, electron transfer (Equation 1), abstraction of the allylic hydrogen (Equation 2) and radical addition to the conjugated double bonds system (Equation 3) [19]. The occurrence of one or another mechanism depends on the organization level of the reaction system and its polarity and on the carotenoid structure [19]. 


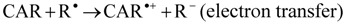
(1)


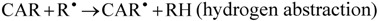
(2)


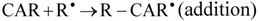
(3)

Astaxanthin was the carotenoid that presented the best scavenging capacity against ROO^•^ and HO^•^. This behavior could be explained by the activation of the hydroxyl groups, due to a possible balance between the keto and enol forms of astaxanthin, which would result in the formation of an ortho-dihydroxy-conjugate polyene system acting as a chain-breaking antioxidant in a similar way to that of α-tocopherol [12].

Although not a radical as HO^•^ and ROO^•^, the reactive species HOCl is a potent oxidant agent that can either act as an antimicrobial agent or cause tissue damages [29]. Moreover, the HOCl has been associated to the development of diverse pathological conditions, such as inflammatory diseases, atherosclerosis, respiratory discomfort, acute vasculitis, rheumatoid arthritis, glomerulonephritis and cancer [30,31]. The HOCl is enzymatically generated *in vivo* by myeloperoxidase (MPO), which uses the H_2_O_2_ produced during the respiratory burst to catalyze the chloride ions (Cl^−^) [29]. Pennathur *et al*. [32] proposed a reaction mechanism between lycopene and HOCl in homogeneous system, in which the Cl atom acts as an electrophile and the double bonds as nucleophiles. When the Cl atom is added to the double bond, through a pseudo-secondary carbocation, a stable chloronium ion is generated. Then, the addition of a hydroxide ion occurs, forming a chlorohydrin, which undergoes a SN2-type reaction with the substitution of the Cl, originating a lycopene epoxide. This epoxide can react with another HOCl molecule (deprotonated by Cl^−^), causing the cleavage of a C–C bond, generating an aldehyde. Astaxanthin and fucoxanthin presented high capacities to scavenge HOCl, suggesting that these carotenoids are potential compounds to be used in the prevention of the development of pathological states related to inflammation.

The ONOO^−^ is a reactive nitrogen species formed *in vivo* from superoxide anion (O_2_^•−^) and nitric oxide (NO^•^) and it is a highly reactive oxidant that causes nitration of the aromatic ring of free tyrosine and protein tyrosine residues. Furthermore, the ONOO^−^ was found to induce various forms of oxidative damage such as low-density lipoprotein (LDL) oxidation, lipid peroxidation, and DNA strand breakage [10]. The reaction between the carotenoid and ONOO^−^ is relatively slow and presents first order kinetics. There are two proposed ways of ONOO^−^ scavenging by carotenoids. In the first one, lycopene accepts energy from ONOO^−^ and goes to an excited state (biradical), and while returning to the ground state produces (*Z*)-isomers. In the second one, lycopene directly reacts with ONOO^−^ to produce a dioxetane that cleaves to apo-lycopenals or undergoes methanolysis to yield methoxy-lycopene [33]. Moreover, recently the formation of nitrocarotenoids as a result of the reaction between carotenoids and ONOO^−^ was described [34,35]. 

Especial attention should be paid to the allenic xanthophyll fucoxanthin, a carotenoid mainly produced by brown seaweeds and microalgae, being the most abundant carotenoid in nature, contributing more than 10% of the estimated total carotenoid production [36]. Fucoxanthin has a peculiar structure, quite different from the carotenoids commonly found in nature, due to the presence of an allenic bond (C=C=C) and some oxygenated functional groups, such as hydroxyl, epoxy, carbonyl and carboxyl (Figure 1). Several biological properties have been attributed to fucoxanthin, such as anti-inflammatory, anticancer, anti-obesity, anti-diabetic, anti-angiogenic, anti-malarial and antioxidant [36,37]. Recently, fucoxanthin was identified as the main carotenoid being responsible for the high antioxidant capacity of extracts from 27 species of brown seaweeds [3], and this activity was mainly attributed to the allenic bond [36]. In the present study, fucoxanthin showed to be an efficient ROO^•^, HO^•^ and HOCl scavenger. 

Our *in vitro* findings reinforce the results of some animal studies [38,39] that showed that the protective effects of astaxanthin on the cardiovascular system are related to its capacity to scavenge ROS and RNS. Moreover, in our study, β-carotene showed to be a less potent ROS scavenger than most of the carotenoids studied, corroborating the fact that, despite presenting some anticarcinogenic activity, β-carotene seems to be less effective than other carotenoids, such as astaxanthin, zeaxanthin, lutein and fucoxanthin [40,41].

## 4. Experimental Section

### 4.1. Chemicals and Standards

Standards of (all-*E*)-β-carotene (99.9%), (all-*E*)-astaxanthin (97.4%), (all-*E*)-fucoxanthin (95.0%), (all-*E*)-canthaxanthin (90.0%), α-tocopherol (97.6%), ascorbic acid (99.0%) and cysteine (97.0%) were supplied by Sigma-Aldrich (St. Louis, MO, USA), (all-*E*)*-*lutein (98.8%), (all-*E*)*-*zeaxanthin (97.4%) and (all-*E*)-lycopene (99.9%) were kindly donated by DSM Nutritional Products (Basel, Switzerland). All these compounds were used as received and the purity of the standards was determined by HPLC-DAD. All carotenoids are in (all-*E*) configuration unless stated otherwise. The fluorescent probe 4,4-difluoro-5-(4-phenyl-1,3-butadienyl)-4-bora-3a,4a-diaza-*s*-indacene-3-undecanoic acid (C_11_-BODIPY^581/591^, MW = 504.43 g/mol) was acquired from Invitrogen (Eugene, OR, USA). The chemicals 6-hydroxy-2,5,7,8-tetramethylchroman-2-carboxylic acid (trolox, 99.5%), soybean L-α-phosphatidylcholine (MW ≈ 900 g/mol), α,α′-azodiisobutyramidine dihydrochloride (AAPH), sodium phosphate dibasic, potassium phosphate monobasic, solution sodium hypochlorite with 13% (w/w) available chlorine, 30% (w/w) hydrogen peroxide solution were purchased from Sigma-Aldrich. Chloroform (PA ACS grade) was purchased from Merck (Darmstadt, Germany); dichloromethane (PA ACS grade), from Labsynth (Diadema, SP, Brazil); ferrous chloride (FeCl_2_), from JT Baker (Phillipsburg, NJ, USA) and ethylenediaminetetraacetic acid, from Quemis (Joinville, SC, Brazil). Ultrapure water was obtained from the Millipore system (Billerica, MA, USA). 

### 4.2. Preparation and Characterization of the Liposomes

Fresh solutions of phosphatidylcholine in chloroform (5 mM) and of the probe C_11_-BODIPY^581/591^ in methanol (2 mM) were prepared. To prepare the liposomes, aliquots of these solutions were transferred to a round-bottomed flask in order to achieve final concentrations of 5 mM of phosphatidylcholine and 5 µM of probe in the liposomes. After that, an aliquot of the antioxidant compound solution, to give a final concentration of about 6 mol%, was added. In the case of the method validation, final concentrations from 1 to 8 mol% of trolox, 6 to 51 mol% of cysteine and 1.6 to 12.8 mol% of ascorbic acid were used (Table 2). The antioxidant concentration in mol% was calculated, dividing the number of moles of the antioxidant compound by the number of moles of phosphatidylcholine and multiplying by 100. The carotenoid solution was prepared in dichloromethane, α-tocopherol in ethanol, trolox and quercetin in methanol, and ascorbic acid and cysteine in phosphate buffered saline (PBS) (12 mM, pH 7.4). The mixture was vortexed for 2 min, sonicated for 2 min and finally vortexed for 30 s. The solvent was evaporated (*T* < 30 °C) while rolling the flask in order to deposit a thin lipid film on the flask wall and left overnight in a freeze-dryer to remove any remaining solvent. For hydration, saccharose (final concentration: 26 mM) and PBS were added to the dry film and the multilamellar lipid vesicles (MLV) were produced by vortexing for 5 min and by sonication for 2 min. The MLV were freeze-thawed three times and extruded in a mini-extruder (Avanti Polar Lipids, Alabaster, AL, USA) by passing them 21 times through a 100 nm polycarbonate membrane to obtain the large unilamelar vesicles (LUV), which were called liposomes within the text. Blank liposomes were also prepared without the addition of antioxidants.

**Table 2:** Validation parameters of micro-assays for the ROS and RNS scavenging capacity in liposomes.

Reactive species	Linearity range (mol%)	*R*^2^ ^a^	Slope ^b^	Intercept ^b^
ROO^•^^c^	1.0–8.0	0.96	20.8	4.7
HO^•^^c^	1.0–6.0	0.97	19.3	3.9
HOCl ^d^	6.0–51.0	0.98	0.17	2.3
ONOO^−^^e^	1.6–12.8	0.98	3.5	9.2

^a^ *R*² is the determination coefficient (*p* < 0.05); ^b^ For each analytical curve, an equation *y* = *ax* + *b* was obtained by linear regression, where *y* is the net area under curve (net AUC), a is the slope and b is the intercept; ^c^ Trolox was used as reference; ^d^ Cysteine was used as reference; ^e^ Ascorbic acid was used as reference.

The liposome size and zeta potential were measured in a ZetaSizer Nano (Malvern, United Kingdom). Liposome average diameters were measured by laser light scattering and ranged from 110 to 140 nm. Zeta potential was measured by electrophoretic mobility and the surface charge was approximately zero. 

The carotenoid concentration was determined in the liposomes by mixing 500 µL of liposomes with 3 mL of dichloromethane and vortexing for 1 min. This mixture was transferred to a separation funnel containing dichloromethane/water (1:1, v/v) and after 3 min, the lower phase containing the carotenoid was collected. The dichloromethane was dried under a N_2_ stream, the carotenoid was redissolved in petroleum ether (β-carotene, zeaxanthin, canthaxanthin, fucoxanthin and lycopene), hexane (astaxanthin) or ethanol (lutein) and the concentration was spectrophotometrically (Agilent model 8453, MO, USA) determined using the specific absorption coefficients for each compound [42]. In the liposomes, the final concentrations were 6 mol% of β-carotene, zeaxanthin, canthaxanthin, fucoxanthin and astaxanthin and 5 mol% of lutein and lycopene.

### 4.3. ROS and RNS Scavenging Capacity Assays

#### 4.3.1. Adaptation and Validation of the Methods

The method to determine the ROO^•^ scavenging capacity of hydrophilic and lipophilic compounds in liposomes developed by Zhang *et al*. [18] was adapted to micro-assays to determine the ROO^•^, HO^•^, HOCl and ONOO^−^ scavenging capacities. These methods are based on the loss of the fluorescence at 600 nm of the probe C_11_-BODIPY^581/591^, a fluorescent lipid, due to its oxidation by these reactive species. This probe was chosen since its sensitivity to oxidation was comparable to that of endogenous fatty acids, and the probe was found previously to be sensitive to HO^•^ and ONOO^−^ but not to O_2_^•^^−^, NO^•^, H_2_O_2_, transition metal ions and hydroperoxides *per se* [43]. The modifications consisted of adapting the reagent volumes to carry out the analysis of ROO^•^ scavenging capacity in microplates and in the use of the same probe to assay the capacity to scavenge HO^•^, HOCl and ONOO^−^. 

The assays were carried out in a microplate reader equipped with a thermostat set at 37 °C and dual reagent dispenser (Synergy Mx, BioTek, Winooski, VT, USA) using 96-well black polystyrene microplates (Corning, New York, NY, USA).

For method validation, an analytical curve was constructed with five concentrations of the compound used as reference (Table 2) for each micro-assay. The linearity between the concentration of the reference compound and the net AUC presented a determination coefficient (*R*^2^) higher than 0.95 (*p* < 0.05), within the range of the tested concentrations, for all the reactive species (Table 2). No interactions between the probe and the compounds used as reference were observed and the loss of fluorescence due to probe photo-bleaching was less than 10% (Supplementary Figure S1). Repeatability was evaluated using the relative standard deviations (RSD) between two independent experiments and was within ±10%, indicating that the developed methods are precise.

#### 4.3.2. Peroxyl Radical Scavenging Assay

ROO^•^ was generated by thermodecomposition of AAPH at 37 °C. Reaction mixtures in the wells contained the following reagents at the indicated final concentrations (final volume of 200 µL): 100 µL of liposomes, 84 µL of PBS (12 mM, pH 7.4) and 16 µL of AAPH (40 mM) solution in PBS. The mixture was pre-incubated in the microplate reader for 10 min before AAPH addition. The fluorescence signal was monitored every minute for the emission wavelength at 540 ± 20 nm with excitation at 600 ± 20 nm, until 180 min. Trolox was used as reference to calculate the ROO^•^ scavenging capacity.

#### 4.3.3. Hydroxyl Radical Scavenging Assay

The HO^•^ was generated by the Fenton reaction (Fe^2+^ + H_2_O_2_ → Fe^3+^ + HO^•^ + OH^−^). Five concentrations of H_2_O_2_ (61, 121, 183, 244 and 286 mM) were tested and the concentration of Fe^2+^/EDTA was set at 313 µM:1250 µM. The H_2_O_2_ concentration of 61 mM was chosen in order to achieve about 5% of the initial fluorescence signal in 120 min in the blank assay (no antioxidant in the liposome). The probe oxidation was also evaluated using only H_2_O_2_ (61 mM) and only Fe^2+^/EDTA (313 µM:1250 µM). These compounds (H_2_O_2 _and Fe^2+^/EDTA) were not able to oxidize the probe by themselves, proving that the HO^•^ generated by the Fenton reaction was the only reagent that reacted with the probe (Supplementary Figure S1). 

Reaction mixtures contained the following reactants at the indicated final concentrations (final volume of 250 µL): 100 µL of liposomes, 59 µL of PBS (12 mM, pH 7.4), 25 µL of FeCl_2_/EDTA (313 µM:1250 µM) and 16 µL of H_2_O_2_ (61 mM). The mixture was pre-incubated in the microplate reader for 10 min before FeCl_2_/EDTA and H_2_O_2 _addition. The fluorescence signal was monitored every minute for the emission wavelength at 540 ± 20 nm with excitation at 600 ± 20 nm, until 240 min. Trolox was used as a reference to calculate the HO^•^ scavenging capacity.

#### 4.3.4. Hypochlorous Acid Scavenging Assay

HOCl was prepared by adjusting the pH of a 1% (v/v) solution of NaOCl to 6.2, with 10% H_2_SO_4_ (v/v). The concentration of HOCl was determined spectrophotometrically at 235 nm using the molar absorption coefficient of 100 M^−1^ cm^−1^. Five concentrations of HOCl (6.9, 1.4, 2.1, 2.8 and 4.3 mM) were tested. The HOCl concentration of 2.8 mM was chosen in order to achieve about 10% of the initial fluorescence signal in about 20 min in the blank assay (no antioxidant in the liposome) (Supplementary Figure S1). 

Reaction mixtures contained the following reactants at the indicated final concentrations (final volume of 200 µL): 100 µL of liposomes, 30 µL of PBS (12 mM, pH 7.4) and 70 µL HOCl (2.8 mM). The mixture was pre-incubated in the microplate reader for 10 min before HOCl addition. The fluorescence signal was monitored every minute for 30 min for the emission wavelength at 540 ± 20 nm with excitation at 600 ± 20 nm. Cysteine was used as reference to calculate the HOCl scavenging capacity.

#### 4.3.5. Peroxynitrite Scavenging Assay

ONOO^−^ was synthesized as previously described by Rodrigues *et al*. [22]. Five concentrations of ONOO^−^ (0.5, 50, 100, 250 and 500 μM) were tested. The ONOO^−^ concentration of 100 μM was chosen in order to achieve about 1% of the initial fluorescence signal in 50 min in the blank assay (no antioxidant in the liposome) (Supplementary Figure S1).

Reaction mixtures contained the following reactants at the indicated final concentrations (final volume of 200 µL): 100 µL of liposomes, 84 µL of PBS (12 mM, pH 7.4) and 16 µL of ONOO^−^ (100 µM). The mixture was pre-incubated in the microplate reader for 10 min before ONOO^−^ addition. The fluorescence signal was monitored every minute for 180 min for the emission wavelength at 540 ± 20 nm with excitation at 600 ± 20 nm. Ascorbic acid was used as reference to calculate the HOCl scavenging capacity.

#### 4.3.6. Calculation of the Scavenging Capacity

The scavenging capacity of all reactive species was calculated according to Equation 4.


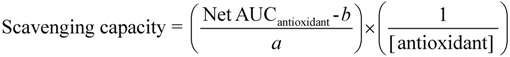
(4)

Where: 


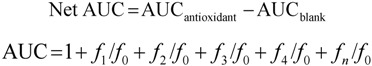


*f*_0_ = initial fluorescence; *f_n_* = fluorescence signal at time *n*; *a* = slope of the analytical curve of [compound used as reference] against Net AUC (Table 2); *b* = intercept of the analytical curve of [compound used as reference] against Net AUC (Table 2); [antioxidant] = concentration of the antioxidant compound in the liposomes (mol%); [compound used as reference] = trolox for ROO^•^ and HO^•^ scavenging capacity, cysteine for HOCl scavenging capacity and ascorbic acid for ONOO^−^ scavenging capacity.

### 4.4. Partition Ratio (log P)

The log *P* values of the carotenoids and other antioxidant compounds (Table 1) were calculated, using water and octanol as solvents, by the ACD/ChemSketch Freeware (version 12.01).

### 4.5. Statistical Analysis

The Software Origin^®^ 8 was used for the calculations. The results were expressed as the mean of two independent experiments (*n* = 2). The analytical curves were plotted by linear regression (*p* < 0.05) and the correlations between the partition ratio (log *P*) and the antioxidant capacity were established using Pearson’s correlation coefficient. 

## 5. Conclusions

Marine carotenoids are compounds with a great potential to scavenge several ROS and RNS at different degrees of efficiency. It is important to highlight that astaxanthin, fucoxanthin, lutein and zeaxanthin were shown to be potent ROS scavengers, whilst β-carotene was the most efficient RNS scavenger. In fact, astaxanthin was shown to be a more potent scavenger of ROO^•^, HOCl and ONOO^−^ than α-tocopherol. The results of the present study reinforce the hypothesis that the antioxidant capacity of the carotenoids is one of the mechanisms responsible for the decrease of the risk of development of cardiovascular diseases and some types of cancer. Moreover, knowledge of the behavior of the carotenoids as antioxidants in lipid bilayers can help the interpretation of the results of *in vivo* studies aiming to correlate the consumption of seafood and seaweeds rich in carotenoids with health benefits. 

## Supplementary Files

Supplementary File 1: PDF-Document (PDF, 241 KB)

## References

[1] Britton G., Britton G., Liaaen-Jensen S., Pfander H. (1995). UV/Visible Spectroscopy. Carotenoids: Spectroscopy.

[2] Mandelli F., Miranda V.S., Rodrigues E., Mercadante A.Z. (2011). Identification of carotenoids with high antioxidant capacity produced by extremophile microorganisms. World J. Microbiol. Biotechnol..

[3] Kelman D., Posner E.K., McDermid K.J., Tabandera N.K., Wright P.R., Wright A.D. (2012). Antioxidant activity of Hawaiian marine algae. Mar. Drugs.

[4] Maoka T. (2011). Carotenoids in marine animals. Mar. Drugs.

[5] Voutilainen S., Nurmi T., Mursu J., Rissanen T. (2006). Carotenoids and cardiovascular health. Am. J. Clin. Nutr..

[6] Zhang J., Dhakal I., Stone A., Ning B., Greene G., Lang N.P., Kadlubar F.F. (2007). Plasma carotenoids and prostate cancer: A population-based case-control study in Arkansas. Nutr. Cancer.

[7] Rock C.L., Britton G., Liaaen-Jensen S., Pfander H. (2009). Carotenoids and cancer. Carotenoids: Nutrition and Health.

[8] Serpeloni J.M., Barcelos G.R.M., Friedmann A., José P., Mercadante A.Z., Bianchi M.L.P., Antunes L.M.G. (2011). Dietary carotenoid lutein protects against DNA damage and alterations of the redox status induced by cisplatin in human derived HepG2 cells. Toxicol. Vitro.

[9] Halliwell B., Gutteridge J. (2007). Radicals in Biology and Medicine.

[10] Valko M., Leibfritz D., Moncol J., Cronin M.T.D., Mazur M., Telser J. (2007). Free radicals and antioxidants in normal physiological functions and human disease. Int. J. Biochem. Cell Biol..

[11] Di Mascio P., Kaiser S., Sies H. (1989). Lycopene as the most efficient biological carotenoid singlet oxygen quencher. Arch. Biochem. Biophys..

[12] Naguib Y.M.A. (2000). Antioxidant activities of astaxanthin and related carotenoids. J. Agric. Food Chem..

[13] Rodrigues E., Mariutti L.R.B., Chisté R.C., Mercadante A.Z. (2012). Development of a novel micro-assay for evaluation of peroxyl radical scavenger capacity: Application to carotenoids and structure-activity relationship. Food Chem..

[14] Müller L., Fröhlich K., Böhm V. (2011). Comparative antioxidant activities of carotenoids measured by ferric reducing antioxidant power (FRAP), ABTS bleaching assay (αTEAC), DPPH assay and peroxyl radical scavenging assay. Food Chem..

[15] Miller N.J., Sampson J., Candeias L.P., Bramley P.M., Rice-Evans C.A. (1996). Antioxidant activities of carotenes and xanthophylls. FEBS Lett..

[16] Roberts W.G., Gordon M.H. (2003). Determination of the total antioxidant activity of fruits and vegetables by a liposome assay. J. Agric. Food Chem..

[17] Woodall A.A., Lee S.W.M., Weesie R.J., Jackson M.J., Britton G. (1336). Oxidation of carotenoids by free radicals: Relationship between structure and reactivity. Biochim. Biophys. Acta.

[18] Zhang J., Stanley R.A., Melton L.D. (2006). Lipid peroxidation inhibition capacity assay for antioxidants based on liposomal membranes. Mol. Nutr. Food Res..

[19] El-Agamey A., Lowe G.M., McGarvey D.J., Mortensen A., Phillip D.M., Truscott T.G., Young A.J. (2004). Carotenoid radical chemistry and antioxidant/pro-oxidant properties. Arch. Biochem. Biophys..

[20] Gruszecki W.I., Landrum J.T. (2009). Carotenoids in Lipid Membranes. Carotenoids: Physical, Chemical, and Biological Functions and Properties.

[21] Faria A.F., Mignone R.A., Montenegro M.A., Mercadante A.Z., Borsarelli C.D. (2010). Characterization and singlet oxygen quenching capacity of spray-dried microcapsules of edible biopolymers containing antioxidant molecules. J. Agric. Food Chem..

[22] Rodrigues E., Mariutti L.R.B., Faria A.F., Mercadante A.Z. (2012). Microcapsules containing antioxidant molecules as scavengers of reactive oxygen and nitrogen species. Food Chem..

[23] Kiokias S., Oreopoulou V. (2006). Antioxidant properties of natural carotenoid extracts against the AAPH-initiated oxidation of food emulsions. Innov. Food Sci. Emerg. Technol..

[24] Scheidegger R., Pande A.K., Bounds P.L., Koppenol W.H. (1998). The reaction of peroxynitrite with zeaxanthin. Nitric Oxide.

[25] Re R., Pellegrini N., Proteggente A., Pannala A., Yang M., Rice-Evans C. (1999). Antioxidant activity applying an improved ABTS radical cation decolorization assay. Free Radic. Biol. Med..

[26] Castro I.A., Rogero M.M., Junqueira R.M., Carrapeiro M.M. (2006). Free radical scavenger and antioxidant capacity correlation of α-tocopherol and Trolox measured by three *in vitro* methodologies. Int. J. Food Sci. Nutr..

[27] Huang D., Ou B., Hampsch-Woodill M., Flanagan J.A., Deemer E.K. (2002). Development and validation of oxygen radical absorbance capacity assay for lipophilic antioxidants using randomly methylated β-cyclodextrin as the solubility enhancer. J. Agric. Food Chem..

[28] Pastor N., Weinstein H., Jamison E., Brenowitz M. (2000). A detailed interpretation of OH radical footprints in a TBP DNA complex reveals the role of dynamics in the mechanism of sequence-specific binding. J. Mol. Biol..

[29] Pullar J.M., Vissers M.C., Winterbourn C.C. (2000). Living with a killer: The effects of hypochlorous acid on mammalian cells. IUBMB Life.

[30] Malech H.L., Gallin J.I. (1987). Neutrophils in human diseases. N. Engl. J. Med..

[31] Malle E., Buch T., Grone H.J. (2003). Myeloperoxidase in kidney disease. Kidney Int..

[32] Pennathur S., Maitra D., Byun J., Sliskovic I., Abdulhamid I., Saed G.M., Diamond M.P., Abu-Soud H.M. (2010). Potent antioxidative activity of lycopene: A potential role in scavenging hypochlorous acid. Free Radic. Biol. Med..

[33] Yokota T., Ohtake T., Ishikawa H., Inakuma T., Ishiguro Y., Terao J., Nagao A., Etoh H. (2004). Quenching of peroxynitrite by lycopene *in vitro*. Chem. Lett..

[34] Yoshioka R., Hayakawa T., Ishizuka K., Kulkarni A., Terada Y., Maoka T., Etoh H. (2006). Nitration reactions of astaxanthin and β-carotene by peroxynitrite. Tetrahedron Lett..

[35] Tsuboi M., Etoh H., Yomoda Y., Kato K., Kato H., Kulkarni A., Terada Y., Maoka T., Mori H., Inakuma T. (2010). Nitration reaction of lutein with peroxynitrite. Tetrahedron Lett..

[36] Peng J., Yuan J.-P., Wu C.-F., Wang J.-H. (2011). Fucoxanthin, a marine carotenoid present in seaweeds and diatoms: Metabolism and bioactivities relevant to human health. Mar. Drugs.

[37] D’Orazio N., Gemello E., Gammone M.A., Girolamo M., Ficoneri C., Riccioni G. (2012). Fucoxantin: A Treasure from the Sea. Mar. Drugs.

[38] Gross G.J., Hazen S.L., Lockwood S.F. (2006). Seven day oral supplementation with Cardax (disodium disuccinate astaxanthin) provides significant cardioprotection and reduces oxidative stress in rats. Mol. Cell Biochem..

[39] Lauver D.A., Driscoll E.M., Lucchesi B.R. (2008). Disodium disuccinate astaxanthin prevents carotid artery rethrombosis and *ex vivo* platelet activation. Pharmacology.

[40] Holick C.N., Michaud D.S., Stolzenberg-Solomon R., Mayne S.T., Pietinen P., Taylor P.R., Virtamo J., Albanes D. (2002). Dietary carotenoids, serum β-carotene, and retinol and risk of lung cancer in the alpha-tocopherol, beta-carotene cohort study. Am. J. Epidemiol..

[41] Nishino H., Murakosh M., Ii T., Takemura M., Kuchide M., Kanazawa M., Mou X.Y., Wada S., Masuda M., Ohsaka Y. (2002). Carotenoids in cancer chemoprevention. Cancer Metastasis Rev..

[42] Davies B.H., Goodwin T.W. (1976). Carotenoids. Chemistry and Biochemistry of Plant Pigments.

[43] Drummen G.P., van Liebergen L.C., Op Den Kamp J.A., Post J.A. (2002). C_11_-BODIPY^(581/591)^, an oxidation-sensitive fluorescent lipid peroxidation probe: (Micro)spectroscopic characterization and validation of methodology. Free Radic. Biol. Med..

