# Supplementary material for: Scavenging Capacity of Marine Carotenoids against Reactive Oxygen and Nitrogen Species in a Membrane-Mimicking System
Source: Mar Drugs. 2012 Aug 20;10(8):1784–98. doi: 10.3390/md10081784 (PMC3447262; doi:10.3390/md10081784)

Supplementary Materials

Figure S1. Decay curves of the probe C_{11}-BODIPY$^{581/591}$ fluorescence using different concentrations of hydroxyl radical generators ($HO^{\bullet}$) and different concentrations of hypochlorous acid (HOCl) and anion peroxynitrite ($ONOO^{-}$). The red line corresponds to the chosen concentrations.

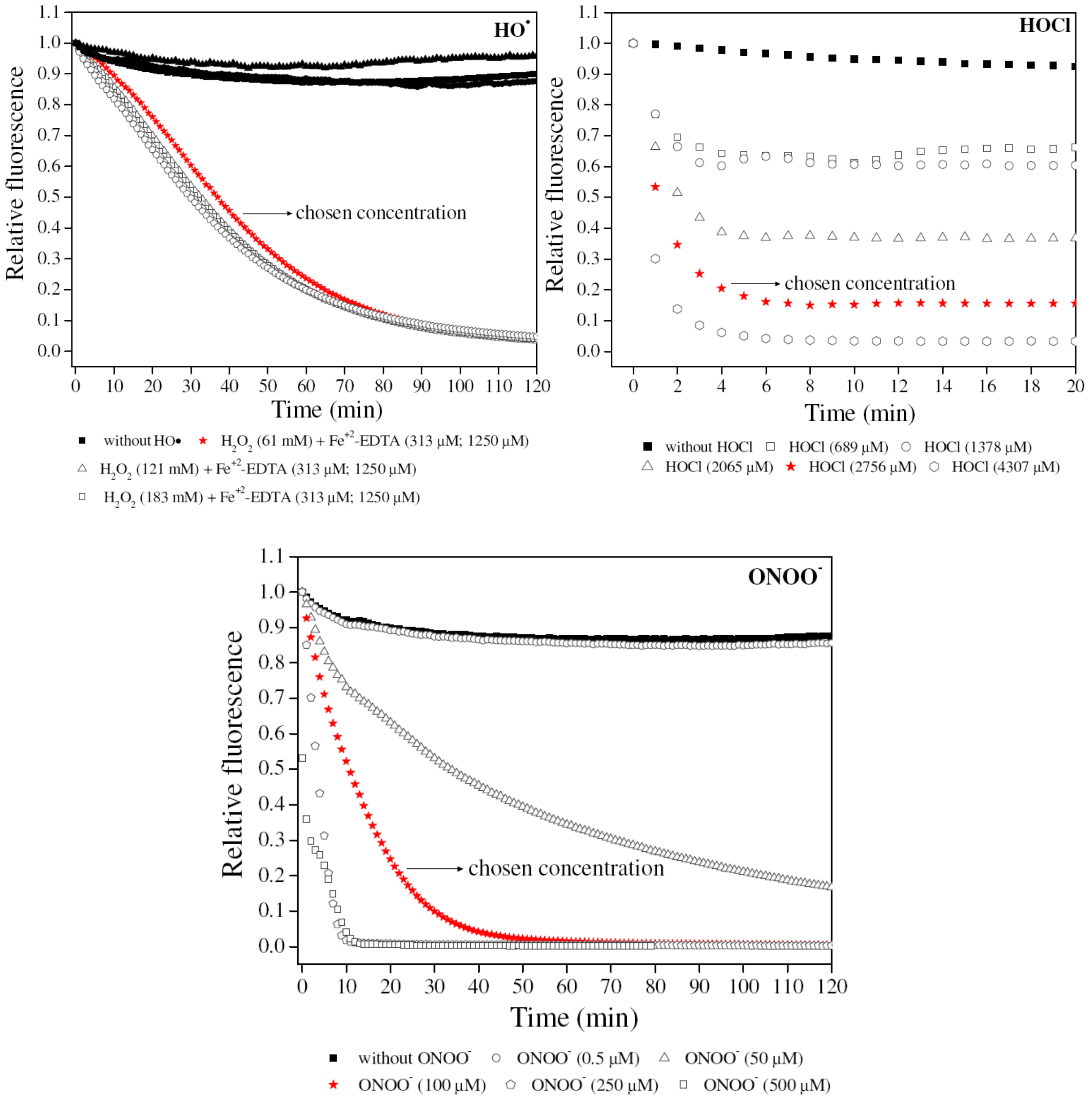

Supplement: Supplementary File 1: — PDF-Document (PDF, 241 KB) [file marinedrugs-10-01784-s001.pdf]
